# Using point-of-care diagnostic testing for improved antibiotic prescription: an economic model

**DOI:** 10.1186/s13561-021-00326-y

**Published:** 2021-08-09

**Authors:** F. Antoñanzas, C. A. Juárez-Castelló, R. Rodríguez-Ibeas

**Affiliations:** Department of Economics, University of La Rioja, La Cigüeña 60, 26004 Logroño, Spain

**Keywords:** Antibiotics, Prescriptions, Diagnostic tests, Infectious diseases, Point of care

## Abstract

**Background:**

Antibiotics have been overprescribed to treat infectious diseases and have generated antimicrobial resistances that reduce their effectiveness. Following the rationale behind the new paradigm of personalized medicine, point-of-care diagnostic testing (POCT) has been proposed to improve the quality of antibiotic prescription with the aim of reducing antimicrobial resistances.

**Methods:**

In order to understand whether this recommendation is valid, we create a theoretical economic model to determine under which conditions the expected benefits of using POCT to guide antibiotic prescription are greater than for empiric prescription, where we define the expected benefits as the difference between the economic value of health and the costs of the treatment. We consider the interaction of a group of physicians who express differing levels of uncertainty when prescribing with a firm selling a diagnostic device, and analyse the firm’s pricing policy and the physicians’ prescribing decisions. We allow the physicians to internalize the external costs of antimicrobial resistances.

**Results:**

We find that the use of POCT reduces the number of antibiotic prescriptions. The reduction in antibiotic prescriptions is higher when physicians internalise the costs of antimicrobial resistances. Physicians with relatively high levels of uncertainty use POCT as they are uncertain about the right treatment for a large proportion of patients. Physicians with low levels of uncertainty prefer to prescribe empirically. The segmentation in the population of physicians regarding the uptake of POCT depends on the distribution of levels of uncertainty across physicians. For each test, the firm charges the marginal production costs of the inputs needed to administer the test, and makes its profit from the sales of the testing devices.

**Conclusions:**

From a theoretical perspective, our findings corroborate the fact that POCT improve the quality of antibiotic prescription and reduce the number of prescriptions. Nevertheless, their use is not always recommended as empiric therapy may be preferred when uncertainty is low.

## Introduction

Effectiveness criteria based on population averages have traditionally guided the choice of treatments for many diseases. These days, however, health care focuses on adapting treatment options to patient characteristics to get the best health outcomes, seeking to avoid prescribing drugs that have no effects on a patients’ health. For many diseases, mainly in the area of oncology, personalized medicine has recently emerged as a new paradigm in health care; the same approach is gaining approval when it comes to antibiotic prescription for infectious diseases.

Physicians have traditionally adopted the empiric prescription of antibiotics; they prescribed antibiotics without knowing whether the treatment was effective against the pathogen. Antibiotics were usually overprescribed, due in part to their low cost, and many patients received unnecessary treatment [1]. Prescriptions in primary care in England increased by 4.1% from 2010 to 2013 [2]. The European Centre for Disease Prevention and Control (ECDC) estimates that 30–50% of all antibiotics prescribed in Europe do not benefit patients. Experts estimate that only 10% of patients with an acute cough who seek medical attention should be prescribed an antibiotic, whereas the actual proportion of prescribed antibiotics in EU countries is 50% overall with a range of between 20 and 80% [3, 4]. Today, the empiric prescription of antibiotics is being challenged due to antimicrobial resistances; the over prescription of antibiotics has caused bacteria to develop resistances, which reduce the therapeutic effects of antibiotics, often eliminating them completely. Bacterial resistances have become a public health problem [5]. Infections with antibiotic-resistant bacteria accounted for 33,110 attributable deaths in the EU and the European Economic Area (EEA) in 2015 ([6]). Adriaenssens et al. [7] found that the quality use of antibiotics had decreased in Europe between 2004 and 2009, promoting the development of antimicrobial resistances. According to the OECD, each year, antimicrobial resistances result in around 1 million disability-adjusted life years (DALYs) lost across the countries of the EU and EEA [8]. There has been recent concern over bacterial resistances and the WHO has called to reduce antibiotic prescription, recommending their use only for infectious diseases of bacterial origin. However, it is not always easy to diagnose the bacterial origin of an infectious disease. It may happen that, according to the observed symptoms, the physician either prescribes antibiotics where not needed or fails to prescribe them where needed.

Antibiotics prescribing causes a negative externality due to the development of antimicrobial resistances. When prescribing, physicians do not internalise the external costs, as they usually do not consider the social costs of these resistances. This behaviour leads to the over prescription of antibiotics. Like in the case of personalised medicine, the use of point-of-care diagnostic testing (POCT) may help and guide antibiotics prescription, as it reduces the uncertainty the physician may have in diagnosing and treating an infectious disease [9]. The use of POCT may partially internalise the negative external costs of the resistances: in the short term, they reduce the number of antibiotic prescriptions; in the medium and long-term, they help to reduce bacterial resistance [10, 11]. For respiratory tract infections, C-reactive protein (CRP) point-of-care tests have been used in several countries in the European Union and their effectiveness has been assessed [2, 12–14]. Researchers have reported that C-reactive protein testing constitutes a cost-effective diagnostic intervention both in terms of reducing antibiotic prescription and in terms of QALYs gained. The cost savings and QALY increase associated with a reduction in infections in the long term outweigh the additional cost per patient of the CRP test. Cooke et al. [15] review the literature on the use of the CRP at point of care to diagnose patients with respiratory tract infections, reporting evidence of a reduction in antibiotic prescription. Huddy et al. [16] studied the pros and cons of adopting CRP testing in primary care in the UK for diagnosing lower respiratory tract infections and concluded that the use of this testing was viable. Lubell et al. [17] also reported that the use of CRP to guide antimicrobial therapy for febrile patients in tropical settings was likely to be cost-effective. In the case of acute tonsillitis, Maizia et al. [18] reported that the use of rapid detection tests for group A Streptococcal was efficient. Rapid tests for pharyngitis in paediatric populations have also proven to be cost-effective ([19–21]).

Although microbiology diagnostic tests are currently used in hospital, they are not usually available in primary care. The adoption of POCT may make sense if the physician is uncertain about the right treatment for a relatively large proportion of patients. The decision to use POCT also hinges upon the costs of testing. In this paper, we present a stylized static theoretical model to analyse, from an economic perspective, the issue of prescribing antibiotics and the adoption of POCT in primary care, identifying the conditions under which physicians will use diagnostic tests to adapt the treatment of infectious diseases. In particular, we model the interaction between a manufacturer of testing devices and a population of physicians who have to decide whether to use POCT when treating their patients. We examine the pricing policy set by the manufacturer and analyse how this affects the decision by physicians to employ POCT. The structure of the article is as follows: in Section 2 below, we describe the model. In Section 3, we include the results. In particular, we determine the expected benefits for prescription decisions made with and without the use of POCT, and segment the population of physicians according to their uptake of POCT. We also characterize the optimal pricing strategy followed by the manufacturer. In Section 4, we discuss the results, and finally, we present some conclusions in Section 5.

## Methods

We consider a population of *N* physicians who treat patients suffering from an infectious disease (for example, a respiratory tract infection). Each physician, in his primary care facility, deals with a group of patients whose size we normalize to one. The patients in each group are indexed by their symptoms *s* ∈ [0, 1], which are uniformly distributed across patients. We can see *s* as an indicator summarizing the patient’s clinical characteristics (age, clinical history, fever, cough, sore throat, etc.) taken into consideration by the physician in deciding on the treatment to pursue.^1^ After the arrival of a patient, the physician observes the symptoms and decides on the treatment. Each patient, depending on the severity of the symptoms, receives either an antibiotic treatment or no medicine.

We assume that antibiotics should be only prescribed when the symptoms are above a certain value *A*, which is unknown by the physicians. For symptoms below *A*, the patient requires no medicine to be cured (self-limiting disease). From the physicians’ perspective, *A* is a random variable whose realization *a* is unknown to the physicians when they decide on the treatment for each patient. Let $\overline{a}\in \left(0,1\right)$ be the expected value of *A*. We may think of $\overline{a}$ as the value of the symptoms above which the prescription of antibiotics is suitable in absence of uncertainty. We assume that the value of $\overline{a}$ is known by all physicians.

Each physician is uncertain about the right treatment for a subset of patients. The cardinality of this subset differs across physicians. For a physician who is uncertain about the right treatment for a subset of patients of cardinality *b*, *A* follows a probability distribution *F*(*A*) in the domain $\left[\overline{a}-0.5b,\overline{a}+0.5b\right]$, with $b\le 2\ \min \left(\overline{a},1-\overline{a}\right)$. Thus, we identify the level of uncertainty of a particular physician as *b*.^2^ A physician with a level of uncertainty *b* prescribes antibiotics to patients with symptoms $s>\overline{a}+0.5b$, gives no treatment to patients with symptoms $s<\overline{a}-0.5b$ and does not know which treatment to prescribe when $s\in \left[\overline{a}-0.5b,\overline{a}+0.5b\right]$. The higher the value of *b*, the more uncertain the physician is. Note that, for a given physician, the proportion of patients whose treatment is uncertain coincides with the width of the range of *A*, i.e. *b*.^3^ For manageability and illustrative purposes, we will assume that *A* is distributed uniformly in the interval [$\overline{a}-0.5b,\overline{a}+0.5b\Big]$, $A\sim U\left(\overline{a}-0.5b,\overline{a}+0.5b\right)$, for $b\in \left[0,2\min \left(\overline{a},1-\overline{a}\right)\right]$ and $\overline{a}\in \left(0,1\right)$.

When deciding on treatments, instead of empiric prescription, each physician may use a diagnostic test (device) at the point of care (POCT). The diagnostic test is marketed by a monopolistic firm that uses a two-part tariff (*C*, *t*), where *C* > 0 is the price of the device and *t* ≥ 0 is the price charged per tested patient.^4^ In our case, *t* is the price for the inputs (reactive) needed to perform the test. The manufacturer charges the same *C* and *t* to all primary care facilities. The diagnostic test determines without error whether the patient needs antibiotics.^5^ We assume, for simplicity, that the unitary production cost of the reactive is zero. Therefore, the profits of the firm are equal to the revenues from the sales of the testing device plus the fees collected from the tests performed. From the perspective of the firm, the level of uncertainty *b* is distributed across physicians according to a density *g*(*b*) with cumulative distribution function *G*(*b*) in $\left[0,2\ \min \left(\overline{a},1-\overline{a}\right)\right]$ with *G*(0) = 0 and $G\left(2\min \left(\overline{a},1-\overline{a}\right)\right)=1$. We assume that *p*^2^ ≤ (*B* + *l*)(*p* − *t*). As we will see later, patients are not tested if *p*^2^ > (*B* + *l*)(*p* − *t*).

When making prescriptions, physicians are aware of the external effects of antimicrobial resistances. We will assume that there is a cost *r* > 0 for each unit of antibiotics given.^6^ When a patient is prescribed antibiotics, the benefit is *B* − *p*_*a*_ − *r* = *B* − *p*, where *B* is the economic value of the health gain (i.e. the price people are willing to pay for good health) and *p*_*a*_ is the price of the antibiotics, *p* = *p*_*a*_ + *r*, *B* > *p*. Due to uncertainty, the physician may prescribe antibiotics when the patient does not need them. In this case, the social benefit is also *B* − *p* as the patient is cured and the treatment must be paid for, but the treatment has no therapeutic effects. If the patient needs antibiotics but the physician does not prescribe them, the patient is not cured and the social benefit is −*l*, where *l* ≥ 0 is the economic value of the disutility suffered by the patient when sick. Finally, when the patient does not require antibiotics and the physician do not prescribe them, the social benefit is *B*. We assume that all patients, regardless of their symptoms, attach the same value to good health, and that the cost of the treatment does not depend on the severity of the symptoms; in other words, each treated patient receives one unit of antibiotics. We assume *B* − *p* ≥ *l*. In the real world, a tested patient may have to attend the primary care facility more than once, and the benefit *B* from the prescribed treatment may otherwise be lower. For simplicity, we assume that *B*, regardless of whether the patient is tested or not, remains the same. Similarly, we consider implicitly that the testing management costs are zero.

Each physician chooses the treatments for her patients to maximize the aggregated expected net health benefits. For each patient, the net health benefits are defined as the difference between the economic value of the health gain (either *B* or −*l*) and the treatment costs (either *p* or zero, plus the testing costs if applied). We assume a perfect agency relationship between the physicians and the primary care facilities as well as between the physicians and the patients. Thus, physicians care about the health outcomes and the treatment costs.

We model the interaction between the firm and the physicians as a two-stage game. In the first stage, the firm chooses (*C*, *t*) to maximize its expected profits. In the second stage, each physician observes (*C*, *t*) and chooses one of the two available strategies. Each physician may choose not to use POCT and decide on the treatments empirically after observing the symptoms of the patients. Alternatively, the physician may decide to use POCT. The physician first decides which patients are tested. These patients are treated according to the results of the test. Non-tested patients are given a treatment similar to the treatment they would have received if the physician had followed empiric prescription. Each physician chooses the strategy that maximizes the aggregated expected net health benefits. We characterize the subgame perfect equilibrium of the game using backward induction; in particular, we find the optimal (*C*, *t*) chosen by the firm and the subset of physicians who use POCT.

## Results

### The second stage: the expected benefit when there is no testing

In this section, we analyse the prescribing behaviour of a physician with a level of uncertainty *b*, $b\in \left[0,2\min \left(\overline{a},1-\overline{a}\right)\right]$, when there is no testing, and find the expected benefit. From now on, we refer to the physician with a level of uncertainty *b* as the *b*-physician. The *b*-physician uses empiric prescription and chooses the patients who will receive antibiotics, i.e. she chooses the level of symptoms $s\in \left[\overline{a}-0.5b,\overline{a}+0.5b\right]$ above which antibiotics are prescribed. To maximize the expected benefit of prescription, the *b*-physician will prescribe antibiotics to patients with symptoms above the symptoms of the patient for whom there is no difference between receiving antibiotics or nothing. Let *s*^∗^(*b*) be the symptoms of the indifferent patient. Symptoms *s*^∗^(*b*) satisfy:

1 $$ B-p=B\left(1-F\left({s}^{\ast }(b)\right)\right)- lF\left({s}^{\ast }(b)\right) $$

The left-hand-side of (1) states the benefit when the patient is prescribed antibiotics. Notice that, regardless of the symptoms, the benefit is *B* − *p*. The right-hand-side of (1) expresses the expected benefit when the patient receives no treatment. The patient experiences a loss *l* with probability *F*(*s*^∗^(*b*)) and is cured with probability 1 − *F*(*s*^∗^(*b*)), where $F\left({s}^{\ast }(b)\right)=\Pr \left(A\le {s}^{\ast }(b)\right)=\frac{s^{\ast }(b)-\overline{a}+0.5b}{b}$. From (1), we get:

$$ \left(B+l\right)F\left({s}^{\ast }(b)\right)=p\Longleftrightarrow \frac{\left(B+l\right)\left({s}^{\ast }(b)-\overline{a}+0.5b\right)}{b}=p\Longleftrightarrow {s}^{\ast }(b)=\overline{a}-0.5b+\frac{pb}{B+l} $$

The *b*-physician prescribes antibiotics to patients with *s* ≥ *s*^∗^(*b*). Let *Q*_*Ae*_(*b*) be the number of antibiotic prescriptions when the *b*-physician follows empiric prescription.

$$ {Q}_{Ae}(b)=1-{s}^{\ast }(b)=1-\overline{a}+0.5b-\frac{pb}{B+l}=1-\overline{a}+\frac{b\left(B+l-2p\right)}{2\left(B+l\right)} $$

The number of antibiotic prescriptions increases with an increase in *b* as long as *B* + *l* > 2*p*. We may expect this condition to be satisfied in real world as *p* is relatively small. From now on, we will assume that this condition is satisfied. Thus, the greater the uncertainty, the higher the number of antibiotic prescriptions. Also, the larger the value of $\overline{a}$, the smaller the number of antibiotic prescriptions. Note that the higher the value of *b*, the lower the value of *s*^∗^(*b*). If *B* + *l* = 2*p*, $s(b)=\overline{a}$ and all physicians, regardless of their level of uncertainty, choose the expected value of *A*.

Let *W*_*e*_(*b*) denote the expected benefit when empiric prescription is used by the *b*-physician. If *s*^∗^(*b*) is larger than the realization of *A*, *s*^∗^(*b*) > *a*, which happens with probability $F\left({s}^{\ast }(b)\right)=\frac{p}{B+l}$, the benefit is *E*(*A*| *s*^∗^(*b*) > *a*)*B* − [*s*^∗^(*b*) − *E*(*A*| *s*^∗^(*b*) > *a*)]*l* + (1 − *s*^∗^(*b*))(*B* − *p*), where $E\left(A|{s}^{\ast }(b)>a\right)={s}^{\ast }(b)-\frac{0.5 pb}{B+l}$ is the expected value of *A* conditioned on *s*^∗^(*b*) > *a*. Patients are divided into three categories depending on the treatment they receive: *E*(*A*| *s*^∗^(*b*) > *a*) patients get no treatment and are cured, [*s*^∗^(*b*) − *E*(*A*| *s*^∗^(*b*) > *a*)] patients should have been prescribed antibiotics but are left untreated, experiencing a loss *l*, and finally 1 − *s*^∗^(*b*) patients are prescribed antibiotics and are cured.

After inserting the value of *E*(*A*| *s*^∗^(*b*) > *a*) in the above expression, we have:

2 $$ \left({s}^{\ast }(b)-\frac{0.5 pb}{B+l}\right)B-\frac{0.5 pb}{B+l}l+\left(1-{s}^{\ast }(b)\right)\left(B-p\right)=B-p\left(1-{s}^{\ast }(b)\right)-0.5 bp $$

If *s*^∗^(*b*) ≤ *a*, which happens with probability $1-F\left({s}^{\ast }(b)\right)=1-\frac{p}{B+l}$, *s*^∗^(*b*) patients are cured without treatment and (1 − *s*^∗^(*b*)) patients receive antibiotics. Some of these patients do not need them. The number of unneeded prescriptions is $\frac{0,5b\left(B+l-p\right)}{B+l}$.

The benefit is:

3 $$ {s}^{\ast }(b)B+\left(1-{s}^{\ast }(b)\right)\left(B-p\right)=B-p\left(1-{s}^{\ast }(b)\right) $$

From (2) and (3), *W*_*e*_(*b*) can be written as:

4 $$ {W}_e(b)=\left(\frac{p}{B+l}\right)\left[B-p\left(1-{s}^{\ast }(b)\right)-0.5 bp\right]+\left(1-\frac{p}{B+l}\right)\left[B-p\left(1-{s}^{\ast }(b)\right)\right]=B-p\left(1-{s}^{\ast }(b)\right)-\frac{0.5b{p}^2}{B+l}=B-p\left(1-\overline{a}\right)-\frac{0.5 pb\left(B+l-p\right)}{B+l} $$

It is easy to see that *W*_*e*_(*b*) decreases with the fall in the level of uncertainty and grows with $\overline{a}$. The next proposition summarizes the results when the *b*-physician does not use the diagnostic test.

**Proposition 1.** *When there is no testing, the b-physician prescribes antibiotics if* $s\ge \overline{a}-0.5b+\frac{pb}{B+l}$*,* $b\in \left[0,2\min \left(\overline{a},1-\overline{a}\right)\right],\overline{a}\in \left(0,1\right).$ *The expected benefit is* $B-p\left(1-\overline{a}\right)-\frac{0.5 pb\left(B+l-p\right)}{B+l}$.

### The second stage: the expected benefit when the b-physician uses POCT

Let us now find the expected benefit when the *b*-physician uses diagnostic testing to decide on the treatments. Let us first determine the subset of patients who are tested. The physician administers the test to a patient as long as there is a benefit.

#### The number of tests

The *b*-physician only uses the test in patients for whom she is uncertain of the treatment to adopt. If a patient with symptoms $s\in \left[\overline{a}-0.5b,\overline{a}+0.5b\right]$ is tested, he will receive antibiotics with a probability $F(s)=\frac{s-\overline{a}+0.5b}{b}$ and no medicine with a probability $1-F(s)=1-\frac{s-\overline{a}+0.5b}{b}.$ The expected benefit is:

5 $$ F(s)\left(B-p\right)+\left(1-F(s)\right)B-t=B- pF(s)-t=B-\frac{p\left(s-\overline{a}+0.5b\right)}{b}-t $$

If the patient is not tested, we know from the previous analysis that the *b*-physician will prescribe antibiotics if *s* ≥ *s*^∗^(*b*) and no medicine otherwise. Let us consider the patients with $s\in \left[{s}^{\ast }(b),\overline{a}+0.5b\right]$. Their benefit is *B* − *p* if they are not tested. From (5), the *b*-physician will administer the test to patients whose symptoms satisfy:

6 $$ B-\frac{p\left(s-\overline{a}+0.5b\right)}{b}-t\ge B-p\Rightarrow \overline{a}+0.5b-\frac{tb}{p}\ge s $$

Let us now consider the patients with $s\in \left[\overline{a}-0.5b,{s}^{\ast }(b)\right)$. We know from (1) that their expected benefit is $B\left(1-F(s)\right)- lF(s)=B-\frac{\left(s-\overline{a}+0.5b\right)\left(B+l\right)}{b}$.

From (5), these patients are tested if:

7 $$ B-\frac{p\left(s-\overline{a}+0.5b\right)}{b}-t\ge B-\frac{\left(s-\overline{a}+0.5b\right)\left(B+l\right)}{b}\Longleftrightarrow s\ge \overline{a}-0.5b+\frac{tb}{B+l-p} $$

Notice that $\overline{a}+0.5b-\frac{tb}{p}\ge {s}^{\ast }(b)\ge \overline{a}-0.5b+\frac{tb}{B+l-p}$ as we are assuming *p*^2^ ≤ (*B* + *l*)(*p* − *t*). If we combine conditions (6) and (7), we conclude that the *b*-physician administers the test to patients with symptoms $s\in \left[\overline{a}-0.5b+\frac{tb}{B+l-p},\overline{a}+0.5b-\frac{tb}{p}\right]$.

**Proposition 2.** *Patients with symptoms* $s\in \left[\overline{a}-0.5b+\frac{tb}{B+l-p},\overline{a}+0.5b-\frac{tb}{p}\right]$ *are tested by the b-physician.*

**Corollary 1.** *If t* = 0*, the test is administered to all uncertain patients.*

From Proposition 2, the number of tests administered by the *b*-physician *T*(*t*, *b*) is

8 $$ T\left(t,b\right)=\left(\overline{a}+0.5b-\frac{tb}{p}\right)-\left(\overline{a}-0.5b+\frac{tb}{B+l-p}\right)=\frac{b\left[\left(p-t\right)\left(B+l\right)-{p}^2\right]}{p\left(B+l-p\right)} $$

It is clear that the number of tests increases with an increase in *b* and decreases with a decrease in *t*.

#### The expected benefit with testing

Let us now find the expected benefit when the *b*-physician uses POCT. We calculate first the gross expected benefit without considering testing costs. When the physician uses POCT, one of the following three situations may occur: 1) all tested patients need antibiotics, 2) all tested patients do not need antibiotics, or 3) some tested patients need antibiotics. We now calculate the gross expected benefit for each situation.

##### All tested patients need antibiotics

**Fig. 1:**
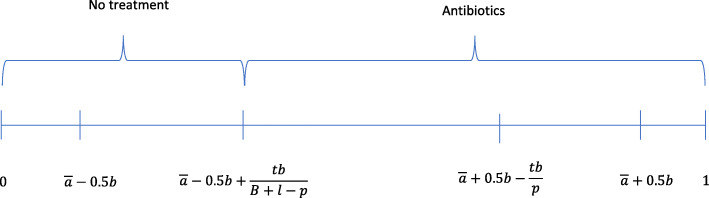
Treatments when the b-physician uses POCT and all tested patients require antibiotics

This situation occurs when the variable *A* takes a value $a\in \left[\overline{a}-0.5b,\overline{a}-0.5b+\frac{tb}{B+l-p}\right)$, which happens with probability ${q}_1=F\left(\overline{a}-0.5b+\frac{tb}{B+l-p}\right)=\frac{t}{B+l-p}$. The conditional expected value of *A* is $\overline{a}-0.5b+\frac{0.5 tb}{B+l-p}$. Patients with $s\in \Big[\overline{a}-0.5b+\frac{0.5 tb}{B+l-p}$, $\overline{a}-0.5b+\frac{tb}{B+l-p}\Big)$ are not tested and receive no treatment even when they should have been prescribed antibiotics. These patients are given a treatment equal to the treatment the *b*-physician would give them without the POCT. They experience an aggregated expected loss equal to $\frac{0.5t\mathrm{b}l}{B+l-p}$. The remaining patients receive the treatments they need. Patients with $s<\overline{a}-0.5b+\frac{0.5 tb}{B+l-p}$ do not require antibiotics and patients with $s\ge \overline{a}-0.5b+\frac{tb}{B+l-p}$ are given antibiotics. Figure 1 depicts the treatments prescribed by the *b*-physician for each symptom level.

Let *EB*_1_(*b*) be the gross expected benefit when all tested patients need antibiotics.

$$ {EB}_1(b)=\left(\overline{a}-0.5b+\frac{0.5 tb}{B+l-p}\right)B-\frac{0.5 tb l}{B+l-p}+\left(1-\overline{a}+0.5b-\frac{tb}{B+l-p}\right)\left(B-p\right)= $$

9 $$ B-p\left(1-\overline{a}\right)-\frac{0.5 tb\left(B+l\right)}{B+l-p}- pb\left(0.5-\frac{t}{B+l-p}\right) $$

If all tested patients need antibiotics, the result is that POCT improves health outcomes. Patients who would have received no treatment otherwise (those whose symptoms $s\in \Big[\overline{a}-0.5b+\frac{tb}{B+l-p}$, *s*^∗^(*b*)]) are tested and, as a result, they are prescribed antibiotics. Antibiotic prescriptions increase with the use of POCT.

##### All tested patients need no treatment

**Fig. 2:**
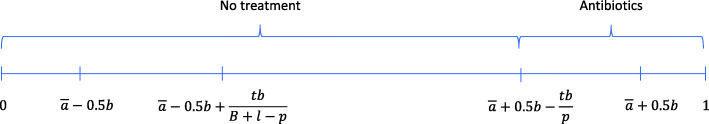
Treatments when the b-physician uses POCT and all tested patients do not require antibiotics

This situation occurs when the variable *A* takes a value $a\ge \overline{a}+0.5b-\frac{tb}{p}$, which happens with probability ${q}_2=1-F\left(\overline{a}+0.5b-\frac{tb}{p}\right)=\frac{t}{p}$. The *b*-physician prescribes nothing to patients with $s\le \overline{a}+0.5b-\frac{tb}{p}$ and prescribes antibiotics to patients with $s>\overline{a}+0.5b-\frac{tb}{p}$. In this case, some patients should not have received antibiotics but the *b*-physician does not have additional information to improve her prescription decision. Figure 2 shows the treatments prescribed by the *b*-physician.

Let *EB*_2_(*b*) be the gross expected benefit when all tested patients need no treatment.

10 $$ {EB}_2(b)=\left(\overline{a}+0.5b-\frac{tb}{p}\right)B+\left(1-\overline{a}-0.5b+\frac{tb}{p}\right)\left(B-p\right)=B-p\left(1-\overline{a}\right)+ pb\left(0.5-\frac{t}{p}\right) $$

When all tested patients need no treatment, the result is that the use of POCT prevents unnecessary antibiotic prescriptions. Patients who otherwise would have been prescribed antibiotics receive no treatment if tested.

##### Only some tested patients need antibiotics

**Fig. 3:**
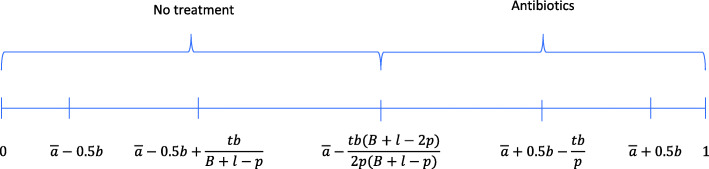
Treatments when the b-physician uses POCT and some tested patients require antibiotics

This situation occurs when the variable *A* takes a value $a\in \left(\overline{a}-0.5b+\frac{tb}{B+l-p},\overline{a}+0.5b-\frac{tb}{p}\right)$, which happens with probability ${q}_3=\left(1-\frac{t}{B+l-p}-\frac{t}{p}\right)$. The conditional expected value of *A* is $\overline{a}-\frac{0.5 tb}{p}+\frac{0.5 tb}{B+l-p}=\overline{a}-\frac{tb\left(B+l-2p\right)}{2p\left(B+l-p\right)}$. Patients with symptoms below this expected value are given no treatment and patients with symptoms above this value are prescribed antibiotics. Figure 3 shows the treatments prescribed by the *b*-physician.

Let *EB*_3_(*b*) be the gross expected benefits when only some tested patients need antibiotics.

$$ {EB}_3(b)=\left(\overline{a}-\frac{tb\left(B+l-2p\right)}{2p\left(B+l-p\right)}\right)B+\left(1-\overline{a}+\frac{tb\left(B+l-2p\right)}{2p\left(B+l-p\right)}\right)\left(B-p\right)= $$

11 $$ B-p\left(1-\overline{a}\right)-\frac{tb\left(B+l-2p\right)}{2\left(B+l-p\right)} $$

In this case, the use of POCT reduces the number of antibiotic prescriptions, as the *b*-physician prescribes no antibiotics to patients with $s\in \left[s\left(\overline{a},b\right),\overline{a}-\frac{tb\left(B+l-2p\right)}{2p\left(B+l-p\right)}\right]$.

Let *EB*(*b*) denote the gross expected benefit when the *b*-physician uses POCT. From (9), (10) and (11), we get:

$$ EB(b)=\sum \limits_{i=1}^3{q}_i{EB}_i(b)=\frac{t}{B+l-p}\left[B-p\left(1-\overline{a}\right)-\frac{0.5 tb\left(B+l\right)}{B+l-p}- pb\left(0.5-\frac{t}{B+l-p}\right)\right]+ $$

$$ \frac{t}{p}\left[B-p\left(1-\overline{a}\right)+ pb\left(0.5-\frac{t}{p}\right)\right]+\left(1-\frac{t}{p}-\frac{t}{B+l-p}\right)\left(B-p\left(1-\overline{a}\right)-\frac{tb\left(B+l-2p\right)}{2\left(B+l-p\right)}\right)=B-p\left(1-\overline{a}\right)-\frac{b{t}^2\left(B+l\right)}{2p\left(B+l-p\right)} $$

Let *W*_*POCT*_(*b*) be the net expected benefit when the *b*-physician uses POCT. By considering the testing costs *tT*(*t*, *b*) from (8) and the price of the testing device *C*, we can write *W*_*POCT*_(*b*) as:

12 $$ {W}_{POCT}(b)= EB(b)- tT\left(t,b\right)-C=B-p\left(1-\overline{a}\right)- tb\left(1-\frac{t\left(B+l\right)}{2p\left(B+l-p\right)}\right)-C $$

The higher the level of uncertainty *b* and the unit cost of the test *t*, the lower the expected benefit. The higher the value of $\overline{a}$, the higher the expected benefit.^7^

$$ \frac{d{W}_{POCT}}{db}=-t\left(1-\frac{t\left(B+l\right)}{2p\left(B+l-p\right)}\right)<0 $$, $$ \frac{d{W}_{POCT}}{dt}=-b\left(1-\frac{t\left(B+l\right)}{p\left(B+l-p\right)}\right)<0 $$, $$ \frac{d{W}_{POCT}}{d\overline{a}}=p>0. $$

Let *Q*_*APOCT*_(*b*) be the expected quantity of antibiotic prescriptions when the *b*-physician uses POCT. From Figs. 1, 2 and 3, we get:

$$ {Q}_{APOCT}(b)=\frac{t}{B+l-p}\left[1-\overline{a}+0.5b-\frac{tb}{\left(B+l-p\right)}\right]+\frac{t}{p}\left[1-\overline{a}-0.5b+\frac{tb}{p}\right]+\left(1-\frac{t}{p}-\frac{t}{B+l-p}\right)\left(1-\overline{a}+\frac{0.5 tb}{p}-\frac{0.5 tb}{B+l-p}\right)=1-\overline{a}+\frac{t^2b\left(B+l\right)\left(B+l-2p\right)}{2{p}^2{\left(B+l-p\right)}^2} $$

The expected number of antibiotic prescriptions *Q*_*APOCT*_(*b*) increases with an increase in *t* and *b*. In section 3.1, we found that the number of antibiotic prescriptions when the *b*-physician does not test the patients was ${Q}_{Ae}(b)=1-\overline{a}+\frac{b\left(B+l-2p\right)}{2\left(B+l\right)}$. According to the reasoning above, the number of antibiotic prescriptions increases when all tested patients need antibiotics but decreases when both all tested or some tested patients need antibiotics. If we compare the number of antibiotic prescriptions for each of the two strategies, we get:

$$ {Q}_{Ae}(b)\gtreqless {Q}_{APOCT}(b)\Longleftrightarrow 1-\overline{a}+\frac{b\left(B+l-2p\right)}{2\left(B+l\right)}\gtreqless 1-\overline{a}+\frac{t^2b\left(B+l\right)\left(B+l-2p\right)}{2{p}^2{\left(B+l-p\right)}^2}\Longleftrightarrow {p}^2{\left(B+l-p\right)}^2\gtreqless {t}^2{\left(B+l\right)}^2\Longleftrightarrow \left(p-t\right)\left(B+l\right)\gtreqless {p}^2 $$

**Proposition 3.** *The b-physician prescribes more antibiotics when the patients are not tested: Q*_*Ae*_(*b*) > *Q*_*APOCT*_(*b*)*, for* $b\in \left[0,2\min \left(\overline{a},1-\overline{a}\right)\right],\overline{a}\in \left(0,1\right).$

### The optimal decision of the *b*-physician

In this subsection, we find the optimal strategy chosen by the *b*-physician. To do so, we compare the expected benefit yielded by both available strategies. From (4) and (12), it follows:

$$ {W}_e(b)\gtreqless {W}_{POCT}(b)\Longleftrightarrow C\gtreqless b\left[\frac{p\left(B+l-p\right)}{2\left(B+l\right)}-t+\frac{t^2\left(B+l\right)}{2p\left(B+l-p\right)}\right] $$

$$ \Updownarrow $$

13 $$ C\gtreqless \frac{b{\left[p\left(B+l-p\right)-t\left(B+l\right)\right]}^2}{2p\left(B+l\right)\left(B+l-p\right)} $$

**Proposition 4.** *The b-physician chooses to use POCT if* $C\le \frac{b{\left[p\left(B+l-p\right)-t\left(B+l\right)\right]}^2}{2p\left(B+l\right)\left(B+l-p\right)}$*.*

Intuitively, we should expect the *b*-type physician to prefer to prescribe without prior testing if the cost of the test *C* is relatively high. Alternatively, the *b*-physician may be more willing to test the patients when her degree of uncertainty (*b*) is relatively high. Note that if a *b*-physician uses POCT, any physician with a level of uncertainty higher than *b* will also use POCT.

### The decision of the firm in the first stage

In the first stage, the firm foresees the behaviour of the physicians in the second stage, and chooses (*C*, *t*) to maximize its expected profits. The firm solves the following problem:

$$ \underset{\left(C,t\right)}{\max }N{\int}_b^{2\min \left(\overline{a},1-\overline{a}\right)}\left[C+ tT\left(t,x\right)\right]g(x) dx $$

$$ s.t\ C\le \frac{b{\left[p\left(B+l-p\right)-t\left(B+l\right)\right]}^2}{2p\left(B+l\right)\left(B+l-p\right)} $$

$$ b\in \left[0,2\ \min \left(\overline{a},1-\overline{a}\right)\right] $$

$$ t\in \left[0,\frac{p\left(B+l-p\right)}{B+l}\right] $$

The firm’s expected profits include the sales of the testing devices plus the fees from the tests performed by the physicians who use POCT. According to the limits of the integral, physicians with levels of uncertainty above *b* use POCT. From Proposition 4, the first constraint states the condition required for the *b*-physician to use POCT. The second constraint states the feasible range of *b*. The last constraint guarantees that testing is performed. Notice that we assumed *p*^2^ < (*B* + *l*)(*p* − *t*) to guarantee testing and this condition can be written as $t<\frac{p\left(B+l-p\right)}{B+l}$.

In the solution to the problem, the first constraint must be binding; otherwise, the firm can increase its profits if it charges a higher price for the device. Therefore, $C=\frac{b{\left[p\left(B+l-p\right)-t\left(B+l\right)\right]}^2}{2p\left(B+l\right)\left(B+l-p\right)}$. If we enter in the objective function this value for *C* and the firm’s revenues from the tests performed $tT\left(t,b\right)= bt\left(1-\frac{t\left(B+l\right)}{p\left(B+l-p\right)}\right)$, we can rewrite the firm’s optimization problems as:

$$ \underset{t}{\max }N{\int}_b^{2\min \left(\overline{a},1-\overline{a}\right)}\frac{x}{2p\left(B+l-p\right)}\left[\frac{{\left(p\left(B+l-p\right)-t\left(B+l\right)\right)}^2}{B+l}+2t\left[\left(p-t\right)\left(B+l\right)-{p}^2\right]\right]g(x) dx $$

*s*. *t* $$ b\in \left[0,2\min \left(\overline{a},1-\overline{a}\right)\right] $$

$$ t\in \left[0,\frac{p\left(B+l-p\right)}{B+l}\right] $$

The derivative of the objective function with respect to *t* is:

$$ N{\int}_b^{2\min \left(\overline{a},1-\overline{a}\right)}\frac{- xt\left(B+l\right)}{p\left(B+l-p\right)}g(x) dx\le 0 $$

The firm’s expected profits decrease with a decrease in *t*. Thus, the firm sets *t* = 0.^8^ The firm wants the physician who uses the testing device to test all the uncertain patients to increase the expected benefit. As the expected benefit is higher, the firm can increase the price for the device.

The firm does not want to distort the testing, leaving some uncertain patients untested. Therefore, the price of the testing device is $C=\frac{bp\left(B+l-p\right)}{2\left(B+l\right)}$. The firm charges the same price *C* to all physicians who use POCT. Finally, the firm chooses *b* to maximize its profits. The problem is:

$$ \underset{b}{\max}\frac{Nbp\left(B+l-p\right)}{2\left(B+l\right)}{\int}_b^{2\min \left(\overline{a},1-\overline{a}\right)}g(x) dx=\frac{Np\left(B+l-p\right)b\left(1-G(b)\right)}{2\left(B+l\right)} $$

The optimal *b*^∗^ is the solution to

$$ 1-G(b)- bg(b)=0 $$

Notice that ${b}^{\ast}\in \left(0,2\min \left(\overline{a},1-\overline{a}\right)\right)$. The firm sells the testing device to all physicians with levels of uncertainty *b* ≥ *b*^∗^ and charges them the same price $C=\frac{b^{\ast }p\left(B+l-p\right)}{B+l}$. The firm sets *t* = 0. Firm’s profits are $\frac{N{b^{\ast}}^2p\left(B+l-p\right)g\left({b}^{\ast}\right)}{B+l}$. From (12), the expected benefit of the physicians who use the testing device is ${W}_{POCT}(b)=B-p\left(1-\overline{a}\right)-\frac{b^{\ast }p\left(B+l-p\right)}{B+l}$, for all *b* ≥ *b*^∗^.

**Fig. 4:**
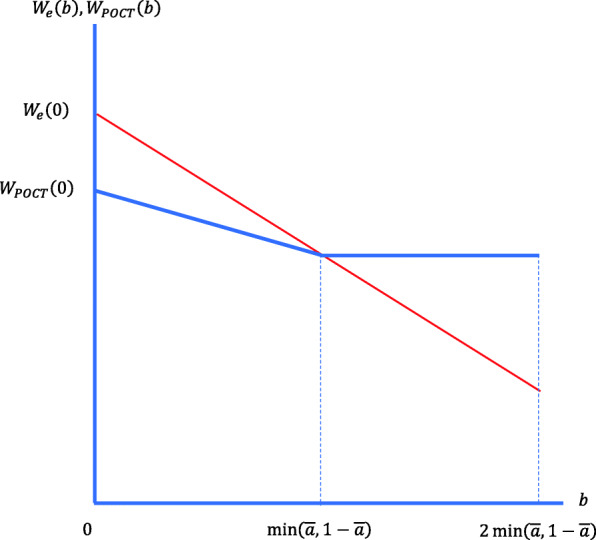
The expected profits of the physicians with uniform distribution of uncertainty

If the level of uncertainty is distributed uniformly across physicians, then ${b}^{\ast }=\min \left(\overline{a},1-\overline{a}\right)$. The firm sells the testing device to physicians with $b\ge \min \left(\overline{a},1-\overline{a}\right)$. The firm chooses as optimum $C=\frac{\min \left(\overline{a},1-\overline{a}\right)p\left(B+l-p\right)}{B+l}$ and *t* = 0. Firm’s profits are $\frac{N\min \left(\overline{a},1-\overline{a}\right)p\left(B+l-p\right)}{2\left(B+l\right)}$. From (12), the expected benefit of the physicians who use the testing device is ${W}_{POCT}(b)=B-p\left(1-\overline{a}\right)-\frac{\min \left(\overline{a},1-\overline{a}\right)p\left(B+l-p\right)}{B+l}$, for all $b\ge \min \left(\overline{a},1-\overline{a}\right)$. Figure 4 illustrates the expected benefits of the physicians and their prescribing behaviour (empiric or test-based) when uncertainty is distributed uniformly. Notice that when there is no uncertainty, *W*_*e*_(0) > *W*_*POCT*_(0) as testing is not needed. For $b\ge \min \left(\overline{a},1-\overline{a}\right)$, *W*_*POCT*_(*b*) > *W*_*e*_(*b*).

**Proposition 5.** *The price of the testing device is* $\frac{b^{\ast }p\left(B+l-p\right)}{B+l}$*, where b*^∗^ *is the solution to* 1 − *G*(*b*) − *bg*(*b*) = 0. *The firm does not charge any fee for each performed test. Only physicians whose level of uncertainty is above b*^∗^ *use POCT.*

**Corollary 2.** *If the level of uncertainty follows a uniform distribution, then the price of the*

*testing device is* $\frac{\min \left(\overline{a},1-\overline{a}\right)p\left(B+l-p\right)}{B+l}$. The physicians who use POCT are those with levels of uncertainty $b\ge \min \left(\overline{a},1-\overline{a}\right)$.

#### Antibiotic prescriptions

We found in Section 3.1 that the number of antibiotic prescriptions when the *b*-physician follows empiric prescribing was ${Q}_{Ae}(b)=1-\overline{a}+\frac{b\left(B+l-2p\right)}{2\left(B+l\right)}$. From Section 3.2, the expected number of antibiotic prescriptions when the *b*-physician tests the patients was ${Q}_{APOCT}(b)=1-\overline{a}+\frac{t^2b\left(B+l\right)\left(B+l-2p\right)}{2{p}^2{\left(B+l-p\right)}^2}$. As *t* = 0, we have ${Q}_{APOCT}(b)=1-\overline{a}$ and ${Q}_{Ae}(b)-{Q}_{APOCT}(b)=\frac{b\left(B+l-2p\right)}{2\left(B+l\right)}$.

When we consider all the physicians who use POCT, the use of POCT reduces the number of antibiotic prescriptions in $\frac{N\left(B+l-2p\right)}{2\left(B+l\right)}{\int}_{b^{\ast}}^{2\min \left(\overline{a},1-\overline{a}\right)} xg(x) dx$.^9^ The reduction in antibiotic prescriptions decreases with *p*. We recall that we had assumed that physicians, when prescribing, consider the costs *r* of the antimicrobial resistances and the price of the antibiotics, *p* = *p*_*a*_ + *r*.

The reduction in antibiotic prescriptions is lower when the physicians do not consider the costs of antimicrobial resistances when prescribing. The number of prescriptions avoided when physicians consider the antimicrobial resistances is $\frac{Nr}{\left(B+l\right)}{\int}_{b^{\ast}}^{2\min \left(\overline{a},1-\overline{a}\right)} xg(x) dx$.

## Discussion

We have analysed, in a context of uncertainty, antibiotic prescription by a group of physicians treating patients displaying symptoms compatible with an infectious disease. Physicians differ in the level of uncertainty experienced when prescribing. We measure uncertainty by considering the proportion of patients for whom the physicians are uncertain about what treatment to apply. Physicians interact with a monopolistic firm that sells a diagnostic device that the physicians can use to reduce the levels of uncertainty and improve the quality of prescription.

Our model does not pursue to explain antibiotics prescription, which is a clinical decision. We carried out an economic decision model to characterize the pricing decision of a diagnostic test manufacturer that sells the testing device to primary care facilities to help prescribe antibiotics and reduce uncertainty. We do not pretend to explain when antibiotics should be prescribed as this decision is a clinical one, and it is based on clinical variables. Our analysis is of economic nature, and its motivation comes from the need to improve antibiotics prescription throughout the use of diagnostic testing (to reduce or eliminate useless antibiotics prescription). To the best of our knowledge, we are not aware of economic models dealing with the clinical decision.

In real world, antibiotics are overprescribed, and antimicrobial resistances have developed. As reported in Cooke et al. [15], 70% of respiratory tract infections are viral, and many others are minor bacterial infections that do not require antibiotics. Antimicrobial resistances constitute a public health problem, insofar as antibiotics are losing their therapeutic effects. To reduce antibiotic prescription, it has been proposed, among other recommendations, to generalize the use of POCT, mainly in primary care, in order to tailor treatments to patients’ clinical characteristics. Diagnostic testing is commonly used in hospitals, but its uptake is still low in primary care facilities and across jurisdictions.

We have developed a stylized static model to illustrate, in a context where physicians are uncertain about the adequate treatment for a subset of patients, whether the use of diagnostic testing to guide antibiotics prescription should be adopted in primary care facilities. The decision to prescribe antibiotics hinges upon the level of uncertainty and the pricing policy set by the monopolistic firm that sells the testing device. We have modelled the interaction between physicians and the firm as a two-stage game where the firm sets the pricing policy in the first stage and the physicians decide in the second stage whether to use the test. The model provides a useful framework for analysing the decision-making processes (patient stratification and treatments) of physicians treating infectious respiratory diseases and the personalisation of treatment. Formally, our analysis is similar to other recent stylized models dealing with personalized medicine. ([23, 24])

We are modelling the pricing decision of the manufacturer assuming that it produces both the testing device and the disposable kit. Alternatively, we could have assumed that the manufacturer sells only the testing device and the disposable kit is available in a competitive market at a price t = 0. In that case, there would be no change in the results.

The model has some limitations. For the sake of simplicity and tractability, we have assumed that the diagnostic testing is perfect. Realistically, the sensitivity and specificity of the test should be lower than 1. Nevertheless, from a qualitative point of view, the results would hold.

We have also assumed that, besides the price of the antibiotics, treatment costs also include the external costs of antimicrobial resistances. Thus, we have assumed that physicians internalise the social costs of antimicrobial resistances when prescribing. This assumption cannot be totally satisfied in real world. A natural extension of the analysis would be to segment the population of physicians in two types depending on whether they actually consider or not these costs. As the model is static, we have assumed that the costs of antimicrobial resistances are exogenous. Furthermore, the social costs of antimicrobial resistances depend on current and future antibiotic prescriptions. A dynamic model would endogenize these costs and analyse how they affect antibiotic prescription. However, this type of model is beyond the scope of our current study.

Finally, we have assumed that physicians behave as perfect agents for both the health care system and the patients. In real world this may not be the case, as physicians may have other interests, what would modify the results of the model; at this point, it is difficult to anticipate the direction of these changes.

## Conclusions

Diagnostic testing may help physicians prescribe treatments when empiric prescription is subject to uncertainty. That is the situation when physicians treat patients suffering from an infectious respiratory disease that may require antibiotics. We have modelled such situation and characterise the conditions under which physicians will use POCTs to guide antibiotic prescription.

We have shown that POCT reduces the number of antibiotic prescriptions. However, the use of POCT is not always the best strategy for all type of physicians, as empiric prescribing can increase the expected benefits (defined as the difference between the expected economic value of health outcomes and the treatment costs) in some cases. When physicians consider the costs of antimicrobial resistances, there is a greater reduction in antibiotic prescriptions.

We have also found that physicians with a sufficiently high level of uncertainty adopt POCT. That level of uncertainty depends on the distribution of the level of uncertainty across physicians.

Regarding the optimal pricing strategy followed by the diagnostics test firm, we found that the firm does not charge for each individual test and make its profits from the sales of the testing device.

In our model, POCT functions as an instrument to internalise in part the negative externality created by prescribing antibiotics. As in the standard economic theory dealing with externalities, we can design a system of incentives to reduce antibiotic prescription, which would work like a subsidy to induce physicians to make the right decision. Physicians may be given monetary bonuses linked to their prescribing behaviour, considering defined daily doses in similar jurisdictions and the health outcomes. As a public policy recommendation derived from our analysis, we can say that public health systems should consider the potential use of POCTs in primary care facilities where clinicians face high levels of uncertainty when prescribing antibiotics. This would be a way to reduce antibiotic consumption maintaining similar health outcomes, and help to cope with antimicrobial resistance issues.

## Data Availability

Not applicable

## Abbreviations

POC: Point-of-care testing
ECDC: European Centre for Disease Prevention and Control
EEA: European Economic Area
OECD: Organisation for Economic Co-operation and Development
DALY: disability-adjusted life year
CRP: C-reactive protein

## Footnotes

1 Butler et al. [4] carry out a clinical study analysing the antibiotics prescription in several primary care facilities for patients suffering from a lower respiratory tract infection. They consider 14 symptoms, and according to them, each patient is given a score between 0 and 100. In our model, each patient has a score between 0 and 1. The doctor observes the symptoms and gives the patient a score. Thus, s in our model is equivalent to the score in Butler et al.’s paper.

2 The level of uncertainty may depend on the physician’s personal characteristics and experience.

3 For example, if $\overline{a}=0.5$, then *b* ∈ [0, 1] and the range of *A* is [0.5(1 − *b*), 0.5(1 + *b*)]. The physician with *b* equal to 1 is uncertain about the treatment for all patients. On the other hand, the physician with *b* equal to 0 prescribes without uncertainty. Similarly, if $\overline{a}=0.7$, *b* ∈ [0,0.6] and the range of *A* is [0.7 − 0.5*b*, 0.7 + 0.5*b*]. A physician with *b* equal to 1 is uncertain about prescribing to patients with *s*∈ [0.2, 0.9].

4 Two-part tariffs are commonly used in practice (telephone, electricity, gas, taxi, etc.).

5 In the real world, the diagnostic tests are not free of error, but their sensitivity and specificity are normally high. For the sake of analytic manageability and simplicity, we assume that there are neither false positives nor false negatives.

6 Shrestha et al. [22] reported that the cost of antimicrobial resistances associated with the consumption of one unit of antibiotics ranged, in the US, from $0.1 for carbapenems to $0.6 for quinolones, cephalosporins and broad-spectrum penicillin.

7 Notice that $$ 1-\frac{t\left(B+l\right)}{p\left(B+l-p\right)}=\frac{\left(p-t\right)\left(B+l\right)-{p}^2}{p\left(B+l-p\right)}>0 $$.

8 If the unit production cost of the inputs to perform the test are positive, the firm would set *t* equal to that marginal cost. The profits come from the sales of the testing device.

9 In the case of uniform distribution, the reduction in antibiotic prescriptions is $\frac{3N\min \left(\overline{a},1-\overline{a}\right)\left(B+l-2p\right)}{4\left(B+l\right)}$.
